# Partial replacement of pork backfat with konjac gel in Northeastern Thai fermented sausage (Sai Krok E-san) to produce the healthier product

**DOI:** 10.5713/ajas.18.0811

**Published:** 2019-03-07

**Authors:** Supaluk Sorapukdee, Sujitta Jansa, Pussadee Tangwatcharin

**Affiliations:** 1 Department of Animal Production Technology and Fisheries, Faculty of Agricultural Technology, King Mongkut’s Institute of Technology Ladkrabang, Bangkok 10520, Thailand

**Keywords:** Sai Krok E-san, Fermented Sausage, Konjac, Low-fat Meat Product, Healthy Meat Product, Functional Meat

## Abstract

**Objective:**

The influence of konjac gel level on fermentation process and product qualities were assessed to evaluate the feasibility of using it as fat analog in Northeastern Thai fermented sausage (Sai Krok E-san).

**Methods:**

Five treatments of fermented sausages were formulated by replacing pork backfat with 0%, 7.5%, 22.5%, and 30% konjac gel. The changes in lactic acid bacteria (LAB) and important physicochemical properties of samples were assessed during 3 days of fermentation. After the end of fermentation at day 3, water activity (a_w_), instrumental texture, color, microbial counts, and sensory evaluation were compared. The best product formulation using konjac for replacing pork back fat were selected and used to compare proximate composition and energy value with control sample (30% pork backfat).

**Results:**

An increase in konjac gel resulted in higher values of LAB, total acidity, and proteolysis index with lower pH and lipid oxidation during 3 days of product fermentation (p< 0.05). It was noted that larger weight loss and product shrinkage during fermentation was observed with higher levels of konjac gel (p<0.05). The resulting sausage at day 3 with 15% to 30% konjac gel exhibited higher hardness, cohesiveness, gumminess, springiness, and chewiness than control (p<0.05). The external color of samples with 22.5% to 30% konjac gel were redder than others (p<0.05). Mold, *Salmonella* spp., *Staphylococcus aureus*, and *Escherichia coli* in all finished products were lower than detectable levels. Product with 15% konjac gel had the highest scores of sourness linking and overall acceptability (p<0.05).

**Conclusion:**

The product with 15% of konjac gel was the optimum formulation for replacing pork backfat. It had higher sensorial scores of sourness and overall acceptability than control with less negative impact on external appearance (product shrinkage) and weight loss. Moreover, it provided 46% fat reduction and 32% energy reduction than control.

## INTRODUCTION

Northeastern Thai fermented sausage, which is called Sai Kork E-san, is a traditional fermented sausage originating from the northeastern part of Thailand and nowadays it is widely consumed in various parts of Thailand. It is also called in Thai as Sai Krok Prew because of its sour taste resulting from meat fermentation by lactic acid bacteria (LAB). The product is roasted and consumed as a main dish together with cooked rice or steamed glutanious rice and thinly sliced fresh or pickled ginger, whole bird chilli, and cabbage. Another similar product, called Nham, has been extensively studied providing the understand of acid meat fermentation [1–5]. These two types of Thai-fermented meat are also formulated from minced pork, cooked rice, garlic, and curing and seasoning ingredients, which are fermented under restrictive oxygen conditions for 2 to 3 days at room temperature, allowing lactic acid fermentation and pH to drop, consequently producing the sour taste. However, there were some differences in recipes and manufacturing processes among products. Northeastern Thai fermented sausage contains a high level of fat and the product mixture is stuffed into natural hog casing prior to hanging for some water drain out, which the final product has a protein content of at least 12% and fat content of 30% or below [6]. On the contrary, Nham is produced from lean meat with pork rind and tightly packaged in plastic casing, where a protein content of at least 20% and fat content of 8% or less are contained in the final product [7]. Currently, consumers more pay attention to the product with lower fat levels due to health concerns such as obesity, colon cancer, and cardiovascular diseases [8]. As reviewed by Grasso et al [9], regarding fat issue, two important strategies to produce healthier meat products include the fat reduction and the modification of fatty acid profile with other healthier fat or oil. Among several ingredients that could be incorporated into a low-fat meat product, konjac-based fat analog is an interesting strategy.

Konjac is a neutral polysaccharide produced from the root of native plant from East Asia, namely *Amorphophallus konjac*, which is utilized as a food additive in Europe with the number of E-425, and classified as Generally Recognized as Safe (GRAS) by the United States Food and Drug Administration (FDA) [10]. Konjac provides numerous technological properties (water-holding capacity and gelling and thickening agents) as well as a potential health implication (dietary fiber, prebiotic, reducing of cholesterol, insulin, and glucose levels or satiating and laxative effects), leading its wide utilization in food products [11]. However, its gelation a with high ability to bind water seems to be the main advantage of konjac flour and it is often used to form gels with other ingredients such as starch, carrageenan, and gellan. Moreover, its gel can be ground to a desired particle size giving the appearance of visible granulated fat and thus is used as a “fat analog” [12]. Konjac gel in different forms and at various concentrations has been used to reduce fat in products such as frankfurters [13,14], bologna [15], fresh sausages [16], pork nuggets [17], Spanish dry-fermented sausage [12], and merguez sausage [10]. However, neither studies regarding konjac gel application for Northeastern Thai fermented sausage, which possess up to 30% fat content, have been reported nor have the changes in qualities during fermentation of both normal- and reduced-fat formulations of these fermented sausages been characterized. Since fat contributes positively to taste, texture, and mouth feeling, how much fat in this distinct sausage can be replaced by konjac gel without a negative impact on sensorial and technological qualities needs to be clarified. Therefore, the objectives of this research were to elucidate the changes in microbiological and physicochemical properties of Northeastern Thai fermented sausage during fermentation when part of the pork backfat was replaced with konjac gel, and to evaluate the sensorial quality, proximate composition, and total energy among different levels of konjac gel replacing pork backfat in the finished product.

## MATERIALS AND METHODS

### Raw material preparation

**Figure 1:**
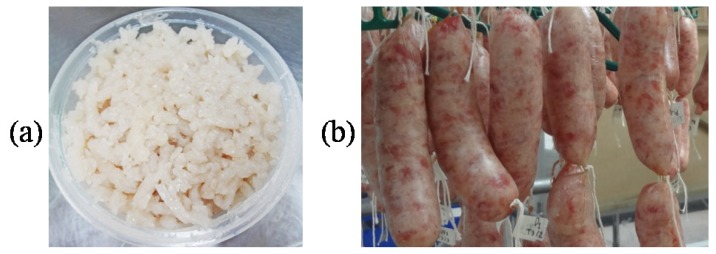
A ground konjac gel used for replacing pork backfat (a). Northeastern Thai fermented sausage containing 15% pork backfat and 15% konjac gel (b).

Fresh pork ham and pork backfat were purchased form a local market. Salted natural hog casing (diameter 28 to 30 mm, Van Hessen, Van Hessen HQ., Nieuwerkerk a/d IJssel, Netherlands) was soaked in water prior to use. Lean ham meat and backfat were separately ground through a 6 mm plate. Konjac gel was prepared as the process described by Osburn and Keeton [14] and Jiménez-Colmenero et al [13]. The ingredients of konjac fat analog were formulated as follows; 5% konjac flour, 1% k-carragenan, 3% corn starch, 0.1% Ca(OH)_2_, and 81% water, which ingredients were kindly donated by Thai Food and Chemical Co., Ltd, Bangkok, Thailand. After forming, the konjac gel was ground through a 6 mm plate and resulting a ground gel as shown in Figure 1a. The ground konjac gel, consisting of approximately 91.5% water, 0.65% ash, 0.39% protein, 0.11% fat, and 5.38% dietary fiber, was used for reformulation of fermented sausage. These raw materials were randomly assigned to five different treatments of fermented sausage.

### Processing of Northeastern Thai fermented sausage and experimental design

Northeastern Thai fermented sausage was prepared as described by Sethakul and Sivapirunthep [18] with slight modifications. Briefly, this sausage consisted of ground pork (55% w/w), ground backfat (30%), and cooked rice (15% w/w) with the following added ingredients (in g/kg of meat mixture): prague powder (15 g/kg, also known as curing salt for meat preservative and composed of 0.90% sodium nitrite and 99.1% sodium chloride), sodium tripolyphosphate (STPP, 3.0 g/kg), sodium erythorbate (2.0 g/kg), monosodium glutamate (2.5 g/kg), sugar (5.0 g/kg), ground garlic (50 g/kg), and fine ground black pepper (4.0 g/kg). To determine the effect of konjac gel when used as pork backfat replacer on product quality, five formulations of mixture were prepared with the following treatments: i) a control treatment (30% backfat without konjac gel); ii) 7.5% konjac gel and 22.5% pork backfat; iii) 15% konjac gel and 15% pork backfat); iv) 22.5% konjac gel and 7.5% pork backfat; v) 30% konjac gel without pork backfat). Each sausage treatment (3 kg/batch) was manufactured as follows. Firstly, meat was mixed with prague powder, STPP, and erythrobate. Then, backfat and/or a konjac gel were mixed followed by cooked rice. The remaining ingredients were added and manually mixed until homogenous. The 65 to 75 g of the prepared sausage mixture was stuffed into natural hog casings using hydraulic sausage stuffer and then hand-linked to sizes of 11 to 12 cm, a picture of the sausage picture after stuffing is shown in Figure 1b. A total of 40 stuffed samples were prepared for each treatment. To start the fermentation process, the stuffed sample was hung at room temperature (32°C±2°C) for 3 days. The manufacturing processes were done in triplicate with 2 weeks intervals, resulting in three independent replication batches (n = 3) for each of the 5 treatment groups (a total of 15 experimental units). Samples from each formulation were taken at day 0, 1, 2, and 3 to monitor the changes in LAB and physicochemical properties. Moreover, after the end of the fermentation at day 3, water activity (a_w_), instrumental texture, color, microbial counts, and sensory evaluation were compared among five formulated treatments. Then the optimum reformulation (based on sensory quality together with physicochemical property) was selected to determined proximate composition and energy value as compared with control sample.

### Changes in lactic acid bacteria and physicochemical properties during 3 days of fermentation

#### Lactic acid bacteria analysis

The samples (25 g) from triplicate sampling were aseptically transferred into 225 mL of sterile saline solution (0.85% w/v NaCl) in a sterile plastic bag. Samples were then homogenized using the Stomacher BagMixers 400 VW (Interscience Co., Saint Nom la Breteche, France). One mL of appropriate dilutions (10^−2^ to 10^−9^) was dropped in duplicate on de Man Rogosa Sharpe (MRS) agar plate supplemented with 0.8% (w/v) calcium carbonate [19]. After anaerobic incubation at 30°C for 24 to 48 h, colonies with clear zones were counted and expressed as logarithms of colony forming units per gram (Log CFU/g).

#### Determination of total acidity

Total acidity of sample was performed according to the method of AOAC [20]. Sample (2 g) was homogenized in 20 mL of distilled water using homogenizer (Ultra-Turrax, IKA Labortechnik, Staufen, Germany). The homogenate was then centrifuged at 5,000× *g* for 15 min and the supernatant was filtered through a filter paper (Whatman No.4, GE Healthcare Thailand, Bangkok, Thailand). Three drops of phenolphthalein solution (1% w/v) was added to the filtrate and titrated with standardized 0.1 N NaOH until a light pink color persisted. Total acidity of the sample was expressed as percentage (%) lactic acid. Triplicate determinations on each treatment were performed.

#### Measurement of pH

The pH of sample was determined by homogenizing 2 g of samples with 20 mL of distilled water using a homogenizer. The pH of suspension was recorded using a combined glass electrode with a digital pH meter (SevenEasy pH meter S20, Mettler Toledo, Schwerzenbach, Switzerland). Triplicate determinations on each treatment were performed.

#### Determination of weight loss and moisture content

Weight loss during fermentation process was evaluated as % of initial weigh of sample (day 0). Moisture content was determined according to AOAC methods [20]. All determinations were performed in triplicate.

#### Determination of trichloroacetic acid-soluble peptides

Trichloroacetic acid (TCA)-soluble peptide contents were determined using the method of Morrissey et al [21]. Ground sample (2 g) was homogenized with 20 mL of 5% (w/v) TCA using an IKA labortechnik homogenizer. The homogenate was kept in ice for 30 min and then centrifuged at 5,000× g for 20 min (Jouan CR3i, Saint-Herblain, France). Soluble peptides in the supernatant were measured by the Lowry method, using tyrosine as standard, and expressed as μmol tyrosine/g sample.

#### Determination of thiobarbituric acid reactive substance

The thiobarbituric acid reactive substance (TBARS) was determined to establish the extent of lipid oxidation and was performed according to the method of Buege and Aust [22]. Briefly, sample (5 g) was dispersed in 25 mL of thiobarbituric acid (TBA) solution containing 0.0375% (w/v) TBA, 15% (w/v) TCA and 0.25 M HCl. The mixture was homogenized for 1 min, heating at 100°C for 10 min and cooled to room temperature with running water. The mixture was centrifuged at the speed of 3,600× *g* for 20 min. The absorbance of the supernatant was read at 532 nm using double beam UV-VIS spectrophotometer (UV-1601, Shimadzu Corporation, Kyoto, Japan). The TBARS value was calculated using a standard curve produced from malonaldehyde bis (dimethyl acetal) (MDA) at concentration ranging from 0 to 10 ppm, and the value was expressed as mg MDA/kg sample. Triplicate determinations were done on each treatment.

### Physicochemical, microbiological, sensorial, and nutritional qualities of finished product

#### Measurement of a_w_

Regarding a_w_ measurement, ground sample was put into water activity pan and a_w_ value was determined using a Novasina LabMaster-a_w_ (Novasina AG., Lachen, Switzerland). Triplicate determinations were done on each treatment.

#### Measurement of texture profile analysis

The sample was subjected to texture profile analysis (TPA) using an Instron universal testing machine model 1011 with a compression plate surface. Six cylinder-shaped samples (~25 mm diameter ×25 mm height) were prepared and placed on the instrument's base. The TPA textural parameters were measured at room temperature with the following testing conditions: crosshead speed was 60 mm/min and compressed twice to 40% of their original height. The Bluehill 2 software (Instron Engineering Corp., Canton, MA, USA) was applied to collect and process the data. The TPA analyses including hardness (N), cohesiveness (ratio), gumminess (N), springiness (ratio) and chewiness (N) were calculated from the force-time curves generated for each sample.

#### Measurement of instrumental color

The external and cross-sectional color of samples were measured by the Commission Internationale de l’Eclairage L*, a*, b* system using a Colorimeter MiniScan EZ 4000L (Hunter Lab Inc, Reston, VA, USA) standardized with a white plate and a black plate. The three replicates of each treatment were taken. Lightness (L*), redness (a*), and yellowness (b*) values were recorded.

#### Microbiological analysis

Microbiological analyses were done in triplicate at day 0 and day 3 of fermentation for yeast and mold, *Staphylococcus aureus* (*S. aureus*), *Salmonella* spp., and *Escherichia coli* (*E. coli*). The samples (25 g) were placed in 225 mL sterile saline solution (0.85% w/v NaCl) in a sterile stomacher bag and were then homogenized using the Stomacher. Serial dilutions were prepared in duplicate and were grown in different culture media. The following media and incubated conditions were used: i) potato dextrose agar (Merck, Darmstadt, Germany) incubated at 25°C for 3 to 5 days for yeast and mold counts, ii) Baird-Parker agar (Merck, Germany) incubated at 37°C for 24 to 48 h and the coagulase test was used for the identification of *S. aureus* colonies for *S. aureus* count [23], and iii) *Salmonella* spp. was estimated in 25 g according to ISO 6579: 2002 [24]. These microbial counts were expressed as logarithms of Log CFU/g. *E. coli* was incubated at 37°C for 24 to 48 h Fluorocult LMX Broth (Merck, Germany) and the IMViC test was used for the identification of *E. coli* to estimate the most probable numbers per gram (MPN/g) [25].

#### Sensory evaluation

The five groups of fermented sausages were prepared for sensory evaluation. Samples were grilled in a pan until the core temperature reached 75°C, as monitored by probes of Type-K thermocouple from a digital thermometer (52 Series II, Fluke Corp., Everett, WA, USA). Cooked samples were evaluated by 12 trained panelists consisting of researchers and meat science graduate students of Department of Animal Production Technology and Fishery, King Mongkut's Institute of Technology Ladkrabang (KMITL). There are 3 sensory evaluation sessions in the present study corresponding to each processing batch and every treatment (5 treatments) was provided in a session. During each evaluation, panelists were served with a half string of sausage from each treatment for evaluating the external appearance, which was determined by visual observation based on product shrinkage and wrinkles on the casing surface. Additionally, panelists were served with 2 cuts of sausages (20 mm thickness) from each treatment for evaluating color, flavor, sourness, texture, juiciness, and overall acceptability of samples. The following nine-point hedonic scale was carried out: 1 = dislike extremely, 2 = dislike very much, 3 = dislike moderately, 4 = dislike slightly, 5 = neither like nor dislike, 6 = like slightly, 7 = like moderately, 8 = like very much, 9 = like extremely. Panelists were served with unsalted cracker and water to refresh their palates before and between samples.

#### Determination of proximate composition and energy value

The best formulations of fermented sausage using konjac for replacing pork back fat were selected and used to analyze proximate composition and energy value as compared with control sample. The proximate compositions (moisture, ash, crude protein, crude fat, dietary fiber, and carbohydrate) of samples were determined in triplicate using the methods described by the AOAC [20]. Total energy content of product was measured by automatic bomb calorimeter (Leco AC-350 calorimeter, Leco Corporation, St. Joseph, MI, USA), which expressed as kcal/100 g. Energy value from fat content (×9 kcal/g) was calculated and reported as a percentage of calories from fat. Total fat and total energy reductions were also calculated.

### Statistical analysis

Statistical analyses for fermented sausage quality during fermentation and finished product quality at the end of fermentation among five treatment of konjac gel were carried out by two-way analysis of variance (ANOVA) and one-way ANOVA, respectively. When a significant effect was found, mean values were compared by the Duncan’s multiple range test. Pearson’s correlation coefficients were evaluated to describe the correlation among parameters. Analysis was performed using the SPSS package (SPSS 16.0 for windows, SPSS Inc., Chicago, IL, USA).

## RESULTS AND DISCUSSION

### Changes in lactic acid bacteria, total acidity, and pH during fermentation

**Figure 2:**
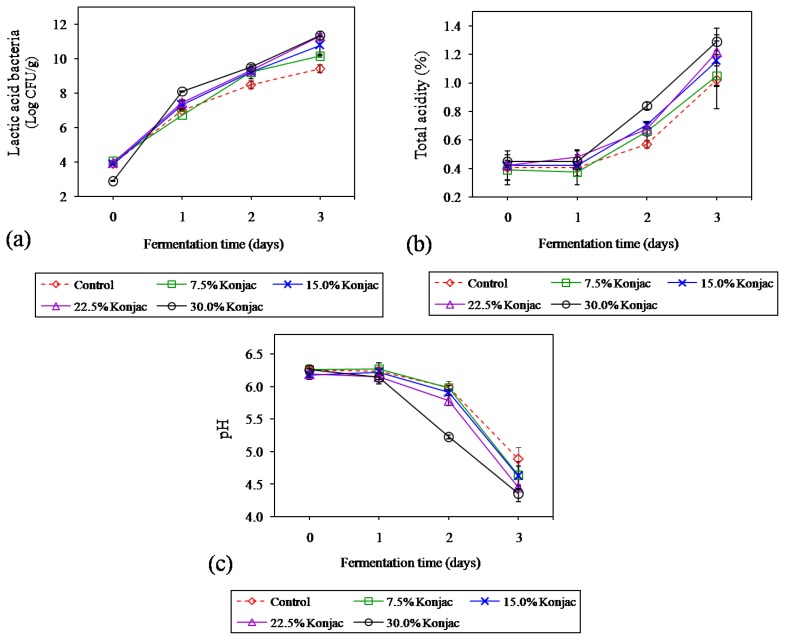
Changes in lactic acid bacteria (LAB) (a), total acidity (b), and pH (c) of Northeastern Thai fermented sausage with different levels of konjac gel during fermentation. Bars represent the standard deviation among triplicate manufacturing of sausages (3 batches, n = 3). Significant differences between treatments and fermentation times were observed in all dependent parameters (p<0.05).

The numbers of LAB in fermented sausages processed by replacing pork backfat with different levels of konjac gel are shown in Figure 2a. The initial levels of LAB in samples at day 0 were 2.91 to 4.07 Log CFU/g. After 3 days of fermentation, these microbial populations increased sharply to 9.42 to 11.38 Log CFU/g, which were higher in samples with higher levels of konjac gel (p<0.05). In sausage formulated with normal fat or a control, the number of LAB were within the range of those found in Nham, where the highest values were 8 to 9 Log CFU/g within the third days of fermentation [5]. Lactobacilli and pediococci are predominant LAB and important for acid production during Nham fermentation [5]. The significantly greater LAB in samples with increased konjac gel (p<0.05) during 3 days of fermentation was probably due to the enhanced growth of bacteria by konjac gel. This evidence was also found in merguez sausage [10], which is discussed later in the results of total acidity and pH.

Results of total acidity and pH are shown in Figure 2b and Figure 2c, respectively. The acidity of the samples increased during fermentation with the highest values at day 3 (1.02% to 1.29%), concomitant with a decrease in the pH to approximately 4.36 to 4.89 within 3 days. These values were similar to another type of fermented meat, Nham, which had a total acidity of 0.77% to 1.60% with pH values ranging from 4.4 to 4.8 within 3 days of fermentation [2]. Glucose and fructose mainly from cooked rice and garlic in Nham formulation were used as the major sources of fermentable carbohydrate by LAB for lactic acid production, resulting in the pH decrease [26]. Visessanguan et al [3] found that among the organic acids detected in Nham, lactic acid was dominantly found as 80% to 90% of the total, followed by acetic and oxalic acid. Furthermore, at the end of fermentation, total acidity was found to be the lowest in control (1.02%±0.02%) and the highest in 30% konjac (1.29%±0.09%) (p<0.05). These results were coincidental with the dramatic decrease of pH, where the largest pH drop was found in 30% konjac (p<0.05). Konjac is polysaccharide-based fiber and mainly composed of mannose separated by glucose units in the backbone, where the glucose to mannose ratio is 1 to 1.6 with one acetyl group per six glucose residues [27]. Konjac provides a prebiotic potential because it shows very little degradation in the digestive tract and is fermented by beneficial human bacterial strains [27,28]. Willaims [11] stated that konjac glucomannan provides a prebiotic addition to the diet, which acts to stimulate the growth of lactic bacteria in the colon and improves fermentation in food processing. Triki et al [10] also found that not only konjac flour but also the pre-gelatinized starch in konjac gel could serve as fermentable carbohydrate sources in a merguez sausage, resulting in lower pH values of formulations with higher levels of konjac gel. Since there was a strong significant correlation of LAB with total acidity (r = 0.820, p<0.01) and pH (r = −0.792, p<0.01) in the present study (data not shown), the increasing of fermentable carbohydrate content implied that replacing pork backfat with a higher content of konjac gel could stimulate the LAB growth, provide the greater extent of lactic acid production and lead to the lower pH.

### Changes in weight loss and moisture during fermentation

**Figure 3:**
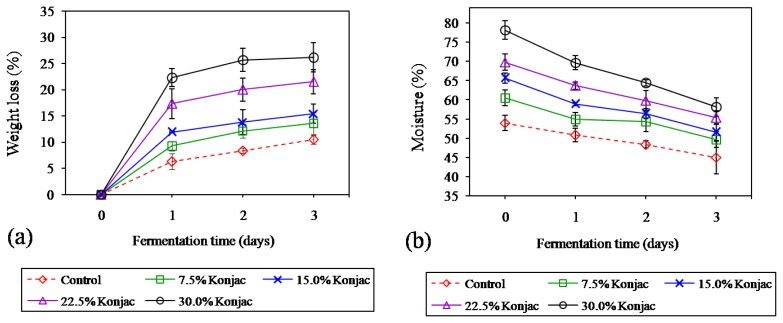
Changes in weight loss (a) and moisture content (b) of Northeastern Thai fermented sausage with different levels of konjac gel during fermentation. Bars represent the standard deviation among triplicate manufacturing of sausages (3 batches, n = 3). Significant differences between treatments and fermentation times were observed in all dependent parameters (p<0.05).

**Figure 4:**
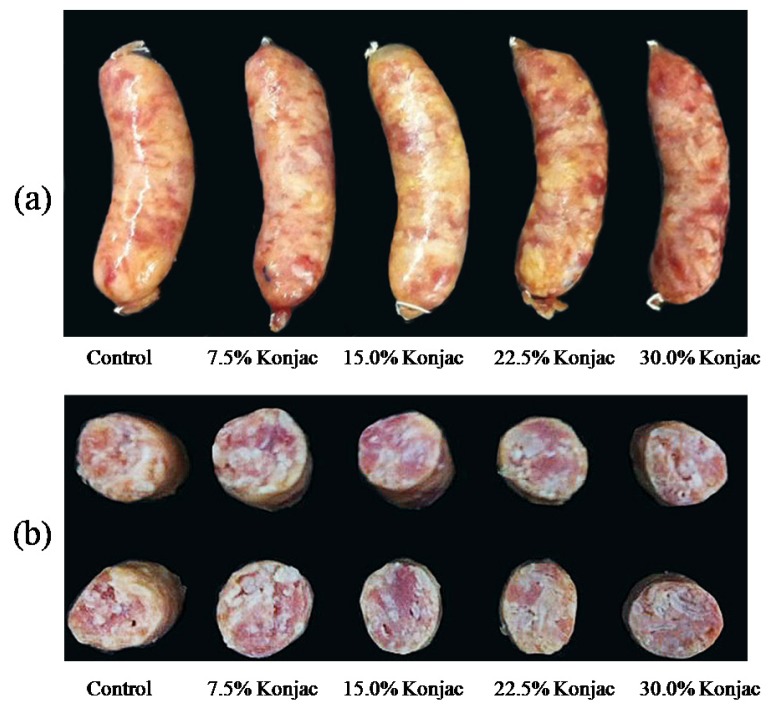
Effect of konjac gel for replacing pork backfat on external (a) and cross-sectional (b) appearances of Northeastern Thai fermented sausage at day 3 of fermentation.

Weight loss of samples was increased during fermentation (p<0.05) as presented in Figure 3a. Consequently, the moisture content of samples decreased as the fermentation time increased (p<0.05) Figure 3b. The extent of water loss during fermentation was mainly dependent on the capacity of meat protein to retain water. Generally, when pH of product during fermentation declines closer to the isoelectric point (pI) of the major proteins (especially for myosin, pI = 5.4 [29]), the net charge of the protein was zero, leading to a reduction in the amount of water was attracted by those proteins. Moreover, partial denaturation of the myosin head at low pH when the temperature is still high is also believed to affect the shrinkage of myofibrillar lattice spacing [29]. This led to an increase in weight loss and a decrease in moisture content of samples during fermentation. Furthermore, increasing the proportion of konjac gel resulted in higher weight loss, but it provided higher moisture content throughout 3 days of fermentation (p>0.05). These results were in agreement with the study of Ruiz-Capillas et al [12], who reported that a dry fermented sausage with more konjac gel provided a higher weight loss, but exhibited a higher water content. Konjac gel used in the current study contained approximately 91% moisture, 0.1% fat, 0.4% protein, 0.6% ash and 5.4% dietary fiber, while Heinz and Hautzinger [30] reported that pork backfat is composed of 7.7% water, 88.7% fat, 2.9% protein, and 0.7% ash. The substitution of pork backfat with an equal amount of konjac gel not only reduced the total fat content of fermented sausage but also increased water added to the product mixture. Moreover, there was a significant negative correlation between weight loss and pH (r = −0.596, p<0.01) (data not shown). These observations emphasize that a greater water loss in a product with 30% konjac gel during fermentation presumably was caused by a higher extent of meat protein denaturation due to rapid pH decline together with some syneresis water loss from konjac gel during processing. Although reformulation with increasing konjac gel could contribute to more water being contained in the product resulting in a high moisture content, the presence of higher weight loss with increasing konjac gel led to a higher level of product shrinkage as shown in Figure 4a, with the highest level of shrinkage occurring in 30% konjac gel added. Unlike water, fat was slightly lost during fermentation process and could be retained in a finished product; therefore, it could retard the shrinkage in a high-fat formulation.

### Changes in trichloroacetic acid-soluble peptides during fermentation

**Figure 5:**
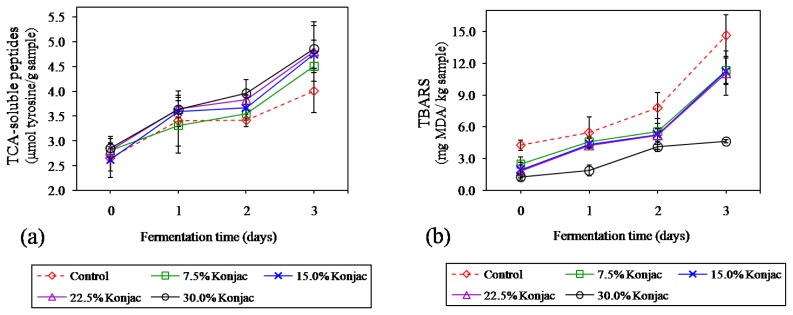
Changes in trichloroacetic acid (TCA)-soluble peptides (a) and thiobarbituric acid reactive substance (TBARS) (b) of Northeastern Thai fermented sausage with different levels of konjac gel during fermentation. Bars represent the standard deviation among triplicate manufacturing of sausages (3 batches, n = 3). Significant differences between treatments and fermentation times were observed in all dependent parameters (p<0.05).

TCA-soluble peptides, an indication of the extent of proteolysis in fermented meat, are shown Figure 5a. It was found that these peptides increased from 2.6 to 2.8 μmol tyrosine/g sample at the beginning to 4.0 to 4.8 μmol tyrosine/g sample at the end of fermentation (p<0.05). Endogenous muscle proteases are considered as having a primary role in proteolysis, while exogenous microbial enzymes from LAB may play a minor role [31], where the extent of proteolysis depends on the product and conditions during ripening [32]. The degradation of myofibrillar and sarcoplasmic proteins was mediated by endogenous cathepsin, followed by the action of bacterial enzymes degrading oligopeptides into small peptides and free amino acids, contributing the flavor and aroma of dry fermented sausages [33] and Nham [4]. Moreover, samples with 15% to 30% konjac added had higher TCA-soluble peptides than control (p<0.05). There was a positive correlation between LAB and these peptides contents (r = 0.908, p<0.01) (data not shown). The results confirmed that konjac formulation could accelerate the fermentation process via stimulating LAB growth as aforementioned, resulting in increasing of small peptides.

### Changes in thiobarbituric acid reactive substance during fermentation

Increasing of TBARS value, indicating increased lipid oxidation during sausage fermentation, was observed in all samples (p<0.05) (Figure 5b). The LAB and other aerobic bacteria isolated from Thai-fermented meat product namely Nham were hydrogen peroxide (H_2_O_2_) producers which is a strong oxidizing agent that can accelerate lipid oxidation and produce a rancid flavor [1]. Visessanguan et al [3,4] stated that although the oxidation level in Nham was relatively high, the levels were not sufficient to produce a detrimental impact on odor or taste in Nham. Stahnke [34] reported that there are several ethyl esters occurring during fermentation of dried sausage which are associated with fruity aromas and can mask rancid odors in the resulting product. Additionally, the control sample showed a higher lipid oxidation than samples with 7.5% to 22.5% konjac, and 30% konjac, respectively (p<0.05). A significant reduction in TBARS value of low-fat merguez sausage by replacing pork backfat with konjac gel has been reported by Triki et al [10]. When pork backfat that possesses high amounts of unsaturated and polyunsaturated fatty acids and is susceptible to oxidation was replaced by konjac gel, the lipid oxidation normally would be minimized. Moreover, there is the strong possibility that replacing pork backfat with konjac gel would contribute to the shelf-stability of the product.

### Finished product characteristics

#### a_w_ and texture

Values of a_w_ among the various formulations were approximately 0.97 to 0.98, which are presented in Table 1, and show that there was no significant differences among samples at the end of fermentation. The a_w_ of fermented meats varies depending on the size of the meat, length of ripening, salt and fat contents, casing permeability, temperature and the relative humidity of the air [35]. An a_w_ value of Nham after the end of fermentation ranged from 0.95 to 0.98 depending on the formulation [36], while levels of a_w_ for dry fermented sausage is around 0.81 to 0.83 [12].

At the end of fermentation, fermented sausages with 15% to 30% konjac gel exhibited higher hardness, cohesiveness, gumminess, springiness, and chewiness as compared to control (p<0.05) (Table 1). In fact, the acid-induced gelation of muscle protein during meat fermentation causes the formation of texture become more rigid, elastic, cohesive, and less adhesive [3]. The more rapid pH decline with increased konjac gel in products might partly associate with the formation of larger aggregation of proteins and resulted in a markedincrease in all texture parameters evaluated by TPA. Similar results have been reported by Ruiz-Capillas et al [12] for dry fermented sausage ripening for 17 days. They stated that increasing of konjac gel to replace fat in dry fermented sausage contributed an increase in hardness and chewiness. However, a decrease in cohesiveness was found as the proportion of konjac gel increased [12]. However, this detrimental effect was not observed in the current study. This is because the composition of the current product was largely different from a dry fermented sausage formulated with 74% pork and 18.5% pork backfat, while Northeastern Thai fermented sausage is produced with 55% pork, 30% pork backfat, and 15% cooked rice. The cooked rice could represent as a binder between meat and konjac parts, therefore, homogenous product was produced and showed a high cohesiveness even though it contained up to 30% konjac gel. Concomitantly with the greater acid-induced gelation with the increasing konjac gel, the higher cohesive texture, especially as compared with control, can be observed in Figure 4b.

### Color

The instrumental color parameters of samples at the end of fermentation are presented in Table 1. While cross-sectional color of samples among various konjac contents (in terms of lightness, redness, and yellowness) were not significantly different (p>0.05), significant differences in redness of the external surface among samples were observed (p<0.05). External parts of samples with 22.5% to 30% konjac gel were redder (higher a* value) than those with 0% to 15% konjac (p<0.05). These results corresponded with product pictures as illustrated in Figure 4a, where the external parts of sausages containing 22.5% to 30% konjac were redder than others (Figure 4b). The redness of the external part of samples with increased konjac gel was related to higher weight loss during fermentation and consequently case hardening. However, the small differences in cross-sectional color might be related to the effect of adding konjac gel, which is a white color and contains a high content of water in the gel. There are inconsistent results regarding the effect of replacing fat with konjac gel among various types of meat products. In dry fermented meat products, the redness tended to decrease with the addition of a higher proportion of konjac gel [12]. In frankfurter sausage, reduced-fat products tended to lower L* and higher a* with increasing konjac gel [13]. Liaros et al [37] suggested that pH, weight loss, fat reduction, and product composition contributed to variations in the myoglobin concentration of fermented sausage and affected to product color.

### Microbiological quality

As previously stated, the LAB was the predominate microorganism in fermented sausage with 9 to 11 Log CFU/g at the 3rd day of fermentation. There were no molds or *Salmonella* spp. detected in initial and finished products (Table 2). *S. aureus* counts were 1 to 2 Log CFU/g at the beginning with a dramatic decrease as konjac gel increased (p<0.05), indicating more were present in pork backfat rather than in konjac gel. However, the population of *S. aureus* declined to a non-detectable level after 3 days of fermentation. *E. coli* were detected only in the control group at the first day and were not found in any samples at the end of fermentation. Enterobacteriaceae and *S. aureus* decreased during 3 days of Nham fermentation as reported by Wiriyacharee [36]. The numbers of yeast were initially found as 3 to 4 Log CFU/g and then decreased to 2–3 Log CFU/g at day 3, with no significant differences between treatments (p>0.05). As summarized by Selgas and Garcia [38], yeasts are most generally found in fresh meat including *Candida*, *Rhodotorula*, *Debaryomyces*, and *Trichosporum*. In fermented meats, although the lactic acid produced by bacteria modified environmental factors that hinder the growth of yeasts, many species can even grow as at pH 4 [39]. Osei Abunyewa et al [40] reported that yeasts were found with initial values of 3 Log CFU/g in commercial salami, but this number increased after 12 days of maturation, reaching a maximum of 5 Log CFU/g at day 20. The presence of yeasts has also been considered to enhance the flavor and aroma of fermented sausage and the *Debaryomyces hansenii* was found to be the dominant yeast observed in fermented sausages with initially 3 to 6 Log CFU/g over 60 days of fermentation, where the final pH value was about 5.7. However, yeast biodiversity in different types of fermented sausages can be varied and also depend upon the pH of final product. In case of the fermented sausage in the present study, the rapid fermentation by LAB producing lactic acid caused the product to attain to pH around 4.6 within 3 days of fermentation, thus retarding the growth of yeasts and pathogenic bacteria.

### Sensory evaluation

The effect of replacing pork backfat with konjac gel in fermented sausage is given in Table 3. The sausage with 22.5% to 30% konjac gel had a lower sensory score of external appearance than the lower levels samples. This meant that the shrinkage of products and the presence of wrinkles on the surfaces of samples containing more than 15% of konjac gel were noticed, leading to a decline in the appearance score which can be seen in Figure 4a. However, samples containing more konjac gel had similar sensory scores of cross-sectional color, texture, and juiciness as compared with control (p>0.05). In some cases, a low-fat dry fermented sausage with 80% konjac gel replacing pork backfat and ripening for 17 days showed a lower juiciness than control [12]. Mendoza et al [41] also reported that the adverse effects of fat reduction in dry fermented sausage were a loss of tenderness and juiciness. In the present study, samples were fermented for a shorter time (3 days) as compared with those reports. Thus, when they formulated with a higher proportion of konjac gel, they still contained higher water content. The release of water fluids from these products could lubricate the texture during chewing, thus the sensation of juiciness and tenderness were not different when compared with the full-fat formulation.

Adding 15% to 30% konjac gel to a product produced a higher flavor scores as compared with control (p>0.05). The favorable intensity of the overall flavor of this kind of fermented sausage was enhanced by fermentation, and acidic and garlic flavors. Due to increased konjac gel, the fermentation process was accelerated, and those intensities were increased, giving a higher score for flavor. Sausage with 15% konjac gel had the highest score for favorable sourness, while excessive sourness in those with 22.5% to 30% could be detected by panelists and resulted in lower score for sourness. Furthermore, the highest overall acceptability was found in the sample with 15% konjac gel, representing the balance formulation for replacing pork back fat as evaluated by sensory testing.

### Proximate analysis and energy value

From the previously result, the product with 15% of konjac gel was the optimum formulation for replacing pork backfat. It provided higher sensory attributes in terms of sourness and overall acceptability than control. It also had a smaller negative impact on external appearance (product shrinkage) and weight loss, as compared with higher levels of konjac gel. When 15% of the pork backfat was replaced with 15% of konjac gel, a low-calorie ingredient with a high content of dietary fiber, fat content in sausage was reduced (Table 4). As the fat content was reduced, moisture, dietary fiber, and carbohydrate contents were greater in the sausages 15% konjac gel than in the control. There were no significant differences in protein and ash contents among samples (p>0.05). These changes represented a fat reduction of around 46%. Not only compositions but also total calorie content and the percentage of calorie from fat are key considerations in designing a new food product [42]. The total energy content of control sample as measured by bomb calorimeter was 339.70 kcal/100 g (around 76% from fat), while the reformulated sample with 15% konjac gel was 228.91 kcal/100 g (around 61% from fat). These changes resulted in total energy reduction around 32.6% as compared with control sample. Similar energy reduction levels were reported in dry fermented sausage [12], fresh pork sausage [14], and merguez sausage [10]. The regulations in Thailand state that the reduced-fat and reduced-total energy products are defined as a minimum of a 25% reduction in fat and total energy as compared with a conventional product [43]. Thus, our developed Northeastern Thai fermented sausage products using 15% konjac gel as fat analog could be considered as the “reduced-fat and reduced-calorie” product, because reductions in fat and total energy were 46% and 33%, respectively, comparing to the conventional formulation.

## CONCLUSION

The sensory evaluation demonstrated that the 15% konjac gel was the optimum content to replace pork backfat in Northeastern Thai fermented sausage, as it provided the highest sourness and overall acceptability scores. This reformulation contained about 46% lower fat content and superior texture and lactic acid production as well as less lipid oxidation than the regular product. It also exhibited less product shrinkage and weight loss during fermentation process as compared to 22.5% to 30% konjac gel.

## Figures and Tables

**Table 1:** Values of a_w_, instrumental texture, and color of Northeastern Thai fermented sausage with different levels of konjac gel

Parameters	0% Konjac (Control)	7.5% Konjac	15.0% Konjac	22.5% Konjac	30.0% Konjac
a_w_	0.97±0.00^a^,1)	0.97±0.01^a^	0.98±0.00^a^	0.97±0.01^a^	0.98±0.00^a^
Instrumental texture					
Hardness (N)	6.51±1.08^c^,1)	7.74±1.13^c^	14.40±1.77^b^	17.84±2.64^a^	18.27±1.21^a^
Cohesiveness (ratio)	0.43±0.01^e^	0.46±0.01^d^	0.52±0.03^c^	0.55±0.01^b^	0.60±0.01^a^
Gumminess (N)	2.82±0.46^c^	3.53±0.58^c^	7.45±0.87^b^	9.85±1.48^a^	10.92±0.79^a^
Springiness (ratio)	0.51±0.01^c^	0.54±0.03^c^	0.67±0.02^b^	0.69±0.03^b^	0.77±0.02^a^
Chewiness (N)	1.44±0.26^d^	1.91±0.29^d^	4.99±0.62^c^	6.81±0.99^b^	8.43±0.78^a^
External color					
Lightness (L*)	45.04±1.87^a^	42.29±2.66^a^	41.29±1.73^a^	42.84±2.61^a^	44.40±2.08^a^
Redness (a*)	4.82±0.64^b^	6.05±0.80^ab^	6.09±0.43^ab^	6.79±0.67^a^	7.11±0.95^a^
Yellowness (b*)	9.17±0.23^a^	8.83±1.13^a^	9.78±0.92^a^	9.25±0.54^a^	8.93±0.53^a^
Cross-sectional color					
Lightness (L*)	46.92±1.94^a^	45.17±3.77^a^	46.14±1.89^a^	46.67±3.26^a^	49.62±2.47^a^
Redness (a*)	4.89±0.40^a^	5.18±1.48^a^	6.07±0.32^a^	6.18±1.37^a^	6.28±0.85^a^
Yellowness (b*)	6.02±0.90^a^	6.49±1.09^a^	6.53±0.28^a^	6.61±0.53^a^	6.87±0.81^a^

1) Values are given as means±standard deviation of each processing batch (n = 3).

a–c Different superscripts in the same row indicate significant differences (p<0.05).

**Table 2:** Microbiological counts of Northeastern Thai fermented sausage with different levels of konjac gel

Microorganisms	Day	0% Konjac (Control)	7.5% Konjac	15.0% Konjac	22.5% Konjac	30.0% Konjac
Yeasts (Log CFU/g)	0	3.24±0.01^a^,1)	3.29±0.04^a^	3.18±0.03^a^	3.20±0.04^a^	3.19±0.03^a^
	3	2.36±0.17^a^	2.43±0.02^a^	2.49±0.08^a^	2.45±0.01^a^	2.49±0.10^a^
Mold (Log CFU/g)	0	<1	<1	<1	<1	<1
	3	<1	<1	<1	<1	<1
*Staphylococcus aureus* (Log CFU/g)	0	2.25±0.03^a^	2.20±0.12^a^	1.97±0.10^b^	1.95±0.07^b^	1.54±0.09^c^
	3	<1	<1	<1	<1	<1
*Escherichia coli* (MPN/g)	0	11	<3	<3	<3	<3
	3	<3	<3	<3	<3	<3
*Salmonella* spp.	0	ND2)	ND	ND	ND	ND
	3	ND	ND	ND	ND	ND

CFU, colony forming units; MPN, most probable numbers.

1) Values are given as means±standard deviation of each processing batch (n = 3).

2) ND, not detect in 25 g.

a–c Different superscripts in the same row indicate significant differences (p<0.05).

**Table 3:** Sensorial scores of Northeastern Thai fermented sausage with different levels of konjac gel

Attributes	0% Konjac (Control)	7.5% Konjac	15.0% Konjac	22.5% Konjac	30.0% Konjac
Appearance	6.54±1.66^a^,1)	6.32±2.10^a^	6.30±1.09^a^	5.16±1.54^b^	4.92±1.55^b^
Color	5.93±1.49^a^	6.77±1.87^a^	6.92±1.25^a^	6.69±1.60^a^	6.77±1.69^a^
Flavor	5.58±1.61^c^	5.96±1.66^bc^	6.88±1.55^ab^	7.27±1.09^a^	7.38±1.12^a^
Sourness	5.81±1.52^b^	6.31±1.09^ab^	7.46±1.19^a^	6.77±1.69^ab^	6.69±1.55^ab^
Texture	5.69±1.89^a^	5.61±1.85^a^	5.76±1.78^a^	5.86±1.71^a^	5.54±1.81^a^
Juiciness	6.27±1.59^a^	6.65±1.49^a^	6.20±1.19^a^	6.46±1.88^a^	6.15±1.22^a^
Overall acceptability	5.54±1.45^b^	5.50±1.87^b^	6.77±1.06^a^	6.59±1.74^ab^	6.46±1.98^ab^

1) Values are given as means±standard deviation of each processing batch (n = 3).

a–c Different superscripts in the same row indicate significant differences (p<0.05).

**Table 4:** Proximate composition and energy values of the different formulations of Northeastern Thai fermented sausage

Parameters	0% Konjac (Control)	15.0% Konjac
Moisture (%)	47.59±0.65^b^,1)	59.43±1.14^a^
Fat (%)	28.72±0.25^a^	15.53±0.35^b^
Protein (%)	13.06±0.06^a^	13.46±0.31^a^
Ash (%)	2.35±0.04^a^	2.58±0.35^a^
Dietary fiber (%)	0.11±0.00^b^	1.42±0.00^a^
Carbohydrates (%)	7.78±0.08^b^	8.89±0.10^a^
Energy value (kcal/100 g)	339.70±3.02^a^	228.91±2.22^b^
Energy from fat (kcal/100 g)	258.48±2.25^a^	139.77±3.15^b^
Energy from fat (%)	76.09±0.66^a^	61.06±1.38^b^
Fat reduction (%)	-	45.9
Energy value reduction (%)	-	32.6

1) Values are given as means±standard deviation of each processing batch (n = 3).

a,b Different superscripts in the same row indicate significant differences (p<0.05).
